# ECM-Mimetic Nylon Nanofiber Scaffolds for Neurite Growth Guidance

**DOI:** 10.3390/nano11020516

**Published:** 2021-02-18

**Authors:** Olga Y. Antonova, Olga Y. Kochetkova, Yuri M. Shlyapnikov

**Affiliations:** Institute of Theoretical and Experimental Biophysics, Russian Academy of Sciences, Pushchino, 142290 Moscow, Russia; office@iteb.ru (O.Y.K.); yuri.shlyapnikov@gmail.com (Y.M.S.)

**Keywords:** nylon scaffolds, nanofibers, neurites growth guidance, focal adhesions

## Abstract

Numerous nanostructured synthetic scaffolds mimicking the architecture of the natural extracellular matrix (ECM) have been described, but the polymeric nanofibers comprising the scaffold were substantially thicker than the natural collagen nanofibers of neural ECM. Here, we report neuron growth on electrospun scaffolds of nylon-4,6 fibers with an average diameter of 60 nm, which closely matches the diameter of collagen nanofibers of neural ECM, and compare their properties with the scaffolds of thicker 300 nm nanofibers. Previously unmodified nylon was not regarded as an independent nanostructured matrix for guided growth of neural cells; however, it is particularly useful for ultrathin nanofiber production. We demonstrate that, while both types of fibers stimulate directed growth of neuronal processes, ultrathin fibers are more efficient in promoting and accelerating neurite elongation. Both types of scaffolds also improved synaptogenesis and the formation of connections between hippocampal neurons; however, the mechanisms of interaction of neurites with the scaffolds were substantially different. While ultrathin fibers formed numerous weak immature β1-integrin-positive focal contacts localized over the entire cell surface, scaffolds of submicron fibers formed β1-integrin focal adhesions only on the cell soma. This indicates that the scaffold nanotopology can influence focal adhesion assembly involving various integrin subunits. The fabricated nanostructured scaffolds demonstrated high stability and resistance to biodegradation, as well as absence of toxic compound release after 1 month of incubation with live cells in vitro. Our results demonstrate the high potential of this novel type of nanofibers for clinical application as substrates facilitating regeneration of nervous tissue.

## 1. Introduction

Various types of peripheral nerve damage are known to be widespread and often lead to loss of sensation, organ, and muscle innervation, which can cause disability [1]. Over the past decade, there have been extensive research and multiple technical innovations in reparative surgery. For instance, in cases of extensive damage, autologous nerve grafts were used to connect the damaged ends of the nerve fibers without tension. The disadvantages of this approach include, but are not limited to, the need for additional surgery; slow rates of regeneration, which can lead to degeneration of target-end-organ after prolonged periods of denervation; inability in some cases to detect the proximal end of the damaged nerve; low numbers of damaged neurons sprouting through the donor tissue, which leads to incomplete restoration of functions and frequent pain [2].

Thereby, efforts are being made to develop synthetic grafts—nerve guidance conduits. However, despite the positive results obtained, their use remains limited to small diastases not exceeding 3 cm. The limitations are related, in particular, to the longer time required to vascularize the longer grafts. This can lead to the formation of an ischemic environment inside the graft and decrease the release of neurotrophic factors due to Schwann cell senescence, which ultimately inhibits axon regeneration. Creating conditions for accelerated growth of damaged axons can be expected to help overcome these limitations [3]. Therefore, the development of new materials that provide high neurite growth rate is a vital challenge requiring a detailed understanding of the cell-surface interaction mechanisms. 

The most straightforward approach to constructing synthetic scaffolds is to mimic the architecture of natural neural ECM [4]. The latter mainly consists of collagen fibers with a diameter of 30–90 nm [5]. Thus, the thin fiber topology is promising for perspective nerve conduits. The most common technique for large-scale production of thin fibers is electrospinning, based on high voltage-induced electrohydrodynamic spraying of various polymer solutions [6]. However, the resulting synthetic scaffold fibers are substantially thicker than the ECM collagen fibers, as shown in Table 1, which summarizes the literature on electrospun scaffolds. The reason for this is the technical limitations of the electrospinning procedure: there have been very few reports of electrospun fibers thinner than 100 nm [7], and to date no such fibers have been used as nerve conduits (see Table 1).

A technique for producing ultrathin nylon-4,6 nanofibers based on electrospinning from a formic acid solution has been reported [22,23]. The diameter of the resulting fibers can be varied, down to approximately 3 nm. Thus, nylon is a unique material among water-insoluble polymers for electrospinning such thin fibers. Its advantages for making scaffolds include good mechanical properties and biodegradability [24]. Although the rate of its degradation in the tissue is low [25], this fact can also be viewed as advantageous, because the scaffold should not be destroyed before the nerve regeneration process is complete. Despite the noted disadvantages of nylon for use in neurosurgery, including high crystallinity and low hydrophilicity [24], in the present work we tried using unmodified nylon for the fabrication of nanofibrous scaffolds for neurite growth guidance. Noteworthy is a recently announced report (doi 10.1016/j.jbiosc.2020.12.003) in which a nylon mesh is also used as an inert material to create novel scaffolds for the adherent culture of neural stem/progenitor cells. Importantly, some published data indicate that the detailed chemical nature of the scaffold surface has little effect on its interaction with cells, since it becomes covered with protein molecules adsorbed from the medium [26], and currently there is no consensus regarding the surface properties which promote neural cell adhesion and neurite growth guidance [13,14].

Various polymers of different chemical nature were used to fabricate electrospun conduits: polycaprolactone (PCL), polyvinyl alcohol, polyvinyl acetate, poly (ethylene oxide), polyethylene glycol, poly (lactic-co-glycolic acid) (PLGA), poly (lactide-co-caprolactone), poly (L-lactic acid) (PLLA), chitosan, cellulose, gelatin and alginate, and for all of them some positive results on neural cell growth were obtained [27]. Table 1 summarizes the quantitative data from the publications which are listed above. However, even for those studies where such data have been reported, a consistent comparison to reveal the best scaffold material is impossible because of different cell models, incubation conditions, and measured quantities. Thus, in this work, we do not claim to have developed the ultimate scaffold material that outperforms all the previously known ones, but we suggest a new direction in the development of synthetic nanostructured conduits. Namely, we propose to use unwoven scaffolds consisting of ultrathin nylon fibers with a diameter comparable to that of the ECM. We compare these scaffolds with the ones composed of thicker nanofibers. We study their interaction with rat hippocampal neurons, measure the total and directional neurite elongation, and characterize the formation of focal contacts of neurons on ultrathin and submicron nylon nanofibers.

## 2. Materials and Methods

Antibodies: anti-integrin beta 1 antibody [P5D2] (Alexa Fluor 647), recombinant anti-synaptophysin antibody [YE269] (Alexa Fluor 488), anti-beta III tubulin antibody [2G10] (Alexa Fluor 488), recombinant anti-vinculin antibody [EPR8185] - Loading control (Alexa Fluor 555), from Abcam (Cambridge, UK). B27 supplement, bovine serum albumin (BSA), calcein-acetoxymethyl (calcein-AM), collagenase type 1 from Life Technologies (Carlsbad, CA, USA), Dil [DilC18(3)] from Invitrogen (Carlsbad, CA, USA), Dulbecco’s modified Eagle’s medium (DMEM) /F12 from Paneco (Moscow, Russia), fetal bovine serum from Gibco (Gaithersburg, MD, USA), 4% formaldehyde in PBS, glycine, glutamine from Paneco (Moscow, Russia), 25 % glutaraldehyde, 98 % formic acid, hexamethyldisilazane, Neurobasal-A medium from Gibco (Gaithersburg, MD, USA), nylon-4,6, penicillin-streptomycin mixed solution, phalloidin-iFluor 488, phalloidin-iFluor 555 from Abcam, PBS from Paneco (Russia), poly-D-lysine, propidium iodide (PI), sodium dodecyl sulfate (SDS), 25% Triton X-100, trypsin, Tween 20. Cell culture 24-well plates, non-cell-adhesive round-bottom 96-well plate, tissue culture flasks from SPL Lifesciences (Pocheon-si, South Korea); 15 mm coverslips from Deltalab (Barselona, Spain). IMR-32 cells were purchased from the Russian Cell Culture Collection (Institute of Cytology RAS, Saint Petersburg, Russia). All other chemicals were purchased from Sigma-Aldrich (Saint Louis, MO, USA), unless otherwise indicated.

### 2.1. Animals

All animal studies were approved by the Animal Ethics Committee of the Institute of Theoretical and Experimental Biophysics RAS.

### 2.2. Preparation of Nanofibrous Scaffolds and Films from Nylon

Free standing nanomats from random ultrathin nylon-4,6 fibers (RU) were fabricated as described earlier [23], by electrospinning from a 10% wt. nylon solution in formic acid. Nanomats from aligned ultrathin (AU) and submicron (AS) fibers with an average diameter of ~60 nm and ~300 nm, respectively, were made by electrospinning from 10% and 20% wt. polymer solutions onto a rotating drum (3.5 cm in diameter with parallel copper wires) at a rotation speed of 2500–5000 rpm. A drum was located 15 cm from the electrospray capillary. To obtain submicron fibers, a spinneret with a diameter of 1 mm was used without additional feeding of formic acid. As in the case of random fibers, ethanol was sprayed simultaneously from a grounded capillary. The resulting free nanomat was transferred to a sterile glass substrate by simple touch. To facilitate the transfer, the copper wires were hydrophobized prior to use by a brief trimethylchlorosilane vapor treatment. The nanomat edges were attached to the substrate by application of polymethyl methacrylate (Plexiglas) solution in benzene followed by drying. When necessary, the sample was further treated with plasma. Electrospinning was carried out in a closed Plexiglas box using pre-sterilized glassware. 

To prepare a film, a 2% nylon solution in formic acid was placed dropwise onto a glass coverslip 15 mm in diameter. The solvent was allowed to evaporate at 37 °C overnight, leaving a flat film on the coverslip.

### 2.3. Morphological Studies of Nylon Nanofibers

Ultrastructural analysis of nylon scaffolds was performed using a Supra 50 VP LEO high-resolution scanning electron microscope with an INCA microanalysis system INCA Energy+ Oxford (LEO Carl Zeiss SMT Ltd, Center of Collective Usage, MSU) at an accelerating voltage of 2 kV. For the analysis, at least five randomly selected fields were taken. Fiber diameter and orientation were quantified using ImageJ software (NIH). To measure the fiber diameter, a line was drawn to span the width of each individual fiber in a given SEM image. The length of each line in pixels was converted to microns using the SEM image scale bar. The fiber diameter was determined as the mean value ± standard error. Fiber orientation was determined using the OrientationJ plugin and Directionality ImageJ software (ver. 153.c, U.S. National Institutes of Health, Bethesda, MD, USA).

To study the scaffold surface, a SmartSPM -1000 atomic force microscope (AIST-NT Co., Moscow, Russia) was also used as described in [9]. Tapping mode with a resonance frequency of 300–350 kHz was employed in all scanning experiments. Optical imaging was performed on a microscope equipped with a dark-field illumination system [28].

### 2.4. Culture of Rat Hippocampal Neurons and Human Neuroblastoma Cells IMR-32 

Hippocampal neurons were derived from the brain of newborn (1–3 days old) Sprague–Dawley rats according to the protocol [29]. Briefly, the hippocampus was dissected and separated from meninges and surrounding tissue prior to enzymatic digestion with 0.25% *w/v* trypsin for 10 min at 37 °C. After pipetting and centrifugation (2000 rpm, 5 min), the resulting cell pellet was resuspended in the hippocampus Neurobasal-A medium and 2% *v/v* B27 solution. Afterwards, 5 × 10^4^ cells were plated side by side on a coverslip coated with 0.1 mg/mL poly-D-lysine, which served as a control, and a nanofiber sample in separate wells of a 24-well plate. Hippocampal neurons were cultivated at 37 °C and 5% *v/v* CO_2_ in a humidified incubator. 

Human neuroblastoma cells IMR-32 were maintained in DMEM/F12 supplemented with 10% fetal bovine serum and a 1% penicillin–streptomycin solution. IMR-32 cells were seeded on nylon nanofibers with a density of 10^5^ cells/cm^2^ and cultured for a predetermined period of time.

### 2.5. Cytotoxicity Test

Cell viability was assessed by incubating the samples in PBS containing 1 µM calcein-AM and 6 μg/mL PI for 15 min at 37 °C and 5% CO_2_. After washing with PBS, the cells were fixed with a 2% formaldehyde solution for 10 min. Confocal laser scanning microscopy was performed to visualize the living and dead cells which produced green and red fluorescence, respectively.

### 2.6. Evaluation of Cell Proliferation during Cultivation on a Nanofiber Scaffold

Evaluation of proliferative activity was carried out according to the number of living cells in experimental and control samples. IMR-32 human neuroblastoma cells were plated onto nylon nanofiber samples in a 24-well plate (10^5^ per well) and incubated for 2, 4, and 6 days. Glass coated with poly-D-lysine was used as a control. Cell counts were assessed using crystal violet assay. Briefly, 1 mL of a 0.5% crystal violet solution was added to each well, followed by incubation in the dark at 37 °C for 20 min. After incubation, the solution was removed, the cells were washed, and the pellet was dissolved in 1% SDS. Three 200 μL replicates from each well were transferred to a 96-well plate for fluorescence reading by a microplate reader Model 680 (Bio-Rad Laboratories, Inc., Hercules, CA, USA). Optical density was recorded at 540 nm. The number of cells was calculated from the previously obtained calibration curve taking into account the sample area.

### 2.7. Scanning Electron Microscope Cell Imaging 

Cells were fixed with 2.5% glutaraldehyde in 0.1 M phosphate buffer, pH 7.2 for 2 h at RT, followed by rinsing with 0.1 M PBS two times. Cells were dehydrated by passing through a series of ethanol solutions, starting at 50% and sequentially incubating in 75%, 80%, 90%, 96%, and 100% ethanol for 5 min, twice repeating each step. The dehydrated cells were incubated twice for 30 min in hexamethyldisilazane. Cells were air dried overnight, coated with chromium for 20 min using a Q150T Turbo-Pumped Sputter Coater (Quorum Technologies) and then imaged with Supra 50 VP LEO (LEO Carl Zeiss SMT Ltd) scanning electron microscope.

### 2.8. Degradation of Nylon Nanofibers

For the in vitro degradation assay, nanofibers adhered to glass were incubated at 37 °C in collagenase type 1 solution (20 U/mg in PBS) for 1 month with a weekly solution change. The samples were then washed, dried, and examined under scanning electron microscopy as described above.

To assess the cytotoxicity of the products of enzymatic cleavage of nylon nanofibers, the samples were incubated in a solution of type 1 collagenase for 1 month with a weekly solution change. To inactivate the enzyme, the collected supernatant was frozen and thawed five times. IMR-32 neuroblastoma cells were seeded at 5·10^4^ cells per well in a 24-well plate. The next day, the resulting supernatant was added to the wells and the cells were cultured for 3 days. Evaluation of cytotoxicity was performed by crystal violet assay as described above, using collagenase type 1 solution as a control. 

### 2.9. Bright-Field Microscopy 

To study the dynamics of morphological changes in neurons, time course imaging was performed using an Axiovert 200 microscope (Carl Zeiss, Göttingen, Germany) with a 40× objective (numerical aperture (NA), 0.3, Ph1). Phase contrast images were obtained after culturing hippocampal neurons on ultrathin and submicron, random and oriented nylon fibers for 1, 2, 5, and 7 days.

### 2.10. Immunochemical Staining

For immunohistochemical staining, cells were fixed with 4% formaldehyde for 10 min at RT, permeabilized in 0.1% Triton X-100 for 5 min and then blocked in 1% BSA, 10% serum, 0.3 M glycine in 0.1% PBS-Tween 20 for 1 h. The cells were then incubated overnight at 4 °C with fluorescent-labeled antibodies: anti-Integrin beta 1 (Alexa Fluor 647) at a working dilution 1:50; recombinant anti-Synaptophysin (Alexa Fluor 488), anti-beta III Tubulin (Alexa Fluor 488) and recombinant anti-Vinculin antibody (loading control - Alexa Fluor 555) at a working dilution 1:100. Following incubation, each well was washed three times with PBS for 5 min. To stain actin, phalloidin-iFluor 488 or phalloidin-iFluor 555 reagent (1:1000) was added to the antibody solution. For membrane staining, cells were incubated with 5 μg/mL Dil for 30 min before adding antibodies. Finally, the cells were mounted on a glass slide and examined under LSCM (Leica TCS SP5, Leica Microsystems CMS GmbH Am Friedensplatz, Mannheim, Germany) using 20× (NA 0.7, oil immersion) and 63× (NA 1.4; oil immersion) objectives. Fiber images were obtained using an argon laser at an excitation wavelength of 488 nm with refraction detection (0.1 pinhole). Experiments were carried out in three or more replicates.

### 2.11. Image Analysis

#### a. Analysis of neuron morphology 

Fields-of-view, randomly selected using confocal microscopy, were analyzed using the NeuronJ semi-automatic tracing tool and Cell counter plugins (NIH ImageJ). The following parameters were investigated: the total length of neurites per cell and the number of cells on the substrate area of 0.25 mm^2^ in comparison with a control glass coated with poly-D-lysine. The length of each neurite was measured from the tip of the neurite to the cell body. This process also made it possible to measure the total length of the neurites per unit area, but since the start and end positions of the neurites could not be determined in each case, only the total length of the neurites was reported. For each case, three independent measurements were performed, in which at least 40 cells were analyzed. The neurite orientation was studied using OrientationJ plugin. The angle of the neurite orientation relative to the fiber direction was determined as the angle that the neurite formed with the nearest fiber or directly in contact with it; the fiber position was taken to be 0°. Neurite was considered "parallel" to the fiber direction if the angles were between 0° and 20°. To measure the neurite diameter, a line was drawn covering the width of each individual neurite and measured with ImageJ instruments. Neurite counts were also performed on the same images using ImageJ, and the number of cells with neurites was calculated.

#### b. Synaptogenesis study

To visualize synaptophysin and the cell membrane, cultured hippocampal neurons were fixed and stained after 7 days of cultivation on samples with antibodies against synaptophysin and Dil, respectively, by confocal microscopy. Images of hippocampal neurons were processed using ImageJ to reduce optical artifacts and thereby improving the detection and quantification of cluster-like signals [30]. After processing, the number of synaptophysin-positive points was counted, their area was measured, and 1-bit masks were created using the “analyze particles” plugin. The length of each neurite was analyzed using the NeuronJ plugin and the number of synaptophysin-positive puncta per 10 μm neurite was calculated. 

#### c. Focal adhesion analysis

After 7 days in culture hippocampal neurons were stained with an anti-Integrin beta-1 antibody, an anti-Vinculin antibody, and Phalloidin. Quantitative assessment of cluster-like signals was performed using the protocol described in [30], similar to the analysis of synaptophysin-positive puncta. For cell area analysis, images were contrasted, a threshold was set, a region of interest was selected at the cell periphery, and cell area was measured. Colocalization of integrin and vinculin clusters was quantified, and Pearson’s correlation coefficient was determined using the Coloc 2 plugin of ImageJ.

### 2.12. Statistics

All experiments were repeated at least three times (n ≥ 3). Experiments with cultured cells included at least three biological replicates. Data are presented as mean ± standard deviation, with the exception of image analysis results which are expressed as mean ± standard error of the mean. A two-sample *t*-test was performed using ANOVA and Student’s *t*-test. In all statistical evaluations, *p* < 0.05 was considered statistically significant. For groups with unequal variances, Mann-Whitney test was used. SigmaPlot 14.0 (Systat Software Inc, San Jose, CA, USA) and Origin 2019b (OriginLab Corporation, Northampton, MA, USA) software were used for statistical analysis.

## 3. Results

### 3.1. Morphological Analysis of Electrospun Nylon Fibers

**Figure 1:**
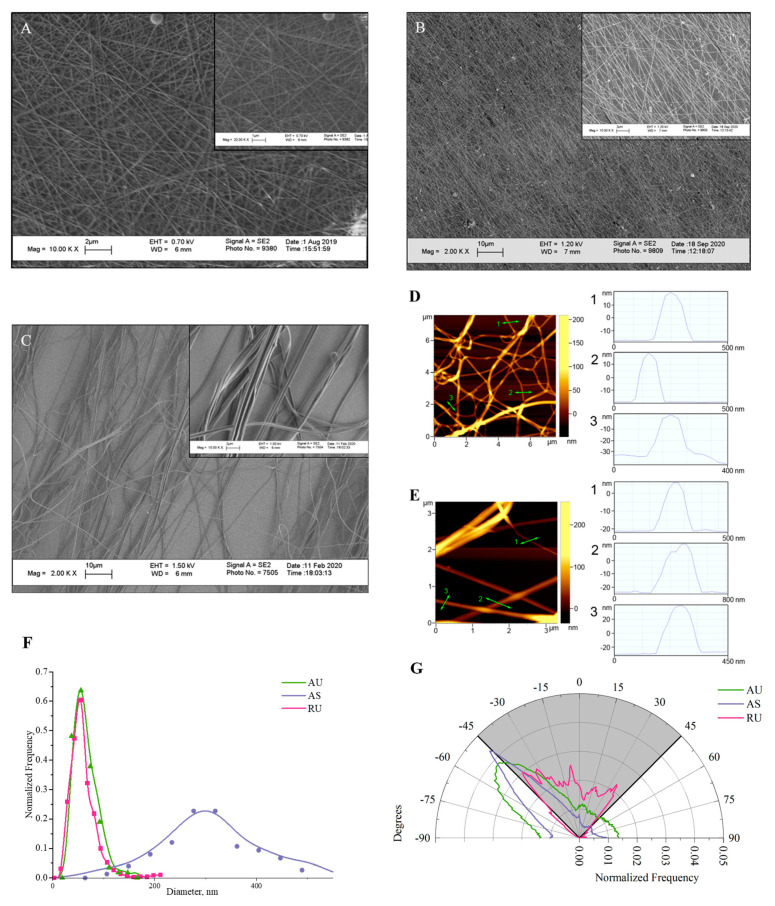
Morphological analysis of nylon nanofibrous scaffolds. SEM images without contrast and histograms of the frequency distribution of the fiber diameter. (**A**)—randomly oriented ultrathin fibers (RU); (**B**)—aligned ultrathin fibers (AU); (**C**)—aligned submicron fibers (AS); (**D**,**E**)—AFM images of RU and AU nanofibers; (**F**)—distribution of nanofiber diameters; (**G**)—angular distribution of fiber direction.

Free-standing nylon nanomats with a random alignment of fibers were prepared by electrospinning a nylon solution in formic acid with gas phase neutralization of the produced positively charged nanofibers by ethanol sprayed from a grounded capillary [23]. Such nanomats have been shown to possess a calibrated pore size [23] meaning that the distance between fibers is not highly variable. To fabricate aligned fibers, we used a common alignment method by depositing the fibers between a set of parallel electrodes on a rotating drum [31]. However, the collection rate of the nylon fibers on the drum was extremely low. Probably, during the electrospinning process, the dielectric screen surrounding the drum (see Experimental section) quickly acquired a positive charge coming from the deposited nanofibers, which electrostatically blocked their collection on the drum. To speed up the collection of fibers, we employed gas phase neutralization of charged fibers, as described in [23]. Using neutralization of the fibers with ethanol, scaffold samples with aligned fibers were produced in ~10 min, similar to the time required for the production of randomly oriented fibers. The spatial orientation and distribution of the resulting nanofiber diameters were investigated by scanning electron microscopy (Figure 1). Electrospinning from a 10% nylon solution was shown to produce ultrathin fibers, both random and oriented, with an average diameter of 60 nm (Figure 1A,B,F). The diameter distribution was narrow: more than 90% of the fibers were thinner than 100 nm. Atomic force microscopy was used as an independent high-resolution method to confirm the diameter values of ultrathin fibers. Although it was possible to scan only a small number of fibers that were in full contact with the mica substrate, AFM confirmed its lowest diameter, which reached 20-30 nm and could be invisible by SEM (Figure 1D,E). The diameter distribution of submicron fibers with an average diameter of 300 nm was broader than that of ultrathin ones (Figure 1C,F), and fibers up to 500 nm diameter were present in the scaffold. Thus, the size of submicron fibers was close to the smallest of the previously studied (Table 1). All obtained nanofibers appeared to be smooth, without a bead-like structure. It should be noted that in this paper we intentionally do not focus on either the spacial or surface density of fibers, but focus on the interaction of cells with individual fibers, depending on their diameter.

For aligned ultrathin fibers, the fiber orientation range was 30°, while AS fibers were oriented in a narrower range of 20° (Figure 1G). As will be shown below, the resulting degree of directionality was sufficient to facilitate coordinated neurite growth. 

### 3.2. Viability and Proliferation of IMR-32 Cells 

**Figure 2:**
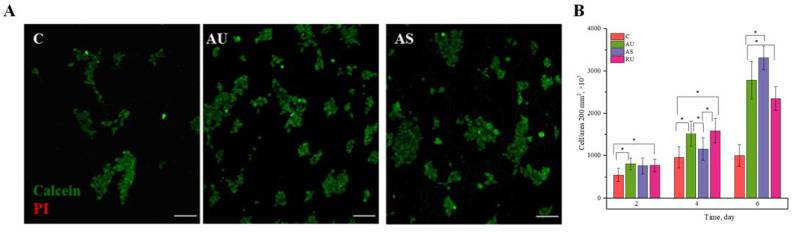
In vitro study of biocompatibility of the experimental scaffolds. (**A**) Representative confocal images of IMR-32 cell viability when cultured on AU and AS fibers for 3 days. Coloring: green–calcein-AM, red–PI. Scale bar—100 µm. (**B**) Proliferation of IMR-32 cells at different times of cultivation. Results from five independent experiments each. C denotes a poly-D-lysine-coated slide used as a control. * Statistically significant difference, *p* < 0.05.

The culture of neuron-like cells of human neuroblastoma IMR-32 was used as a model object to study the cytotoxicity of fabricated nylon scaffolds and their effect on cell proliferation. For quantitative assessment, we seeded IMR-32 cells on nanofiber samples with different ultrastructure and monitored cell viability and proliferation. The surface of the nanofibers was not additionally modified with compounds that promote cell adhesion, such as poly-D-lysine, polyethyleneimine, collagen or laminin, but instead was treated with plasma to enhance the adsorption of proteins from the cell medium [32]. The results obtained by confocal microscopy are shown in Figure 2A and Figure S1. The absence of PI-positive cells indicates that the nylon fiber scaffolds were non-toxic to cells. As seen in Figure 2B, regardless of fiber diameter and direction, nanostructured substrates showed significant increase in cell counts over time: after 6 days of incubation their numbers increased 2 to 3-fold compared to the control samples. There was no statistical difference in the number of cells on scaffolds of aligned and randomly oriented ultrathin fibers by the 6th day of cultivation. Thus, growth of IMR-32 neuroblastoma cells on nylon scaffolds demonstrates their good biocompatibility.

Analysis of biodegradation of nylon fiber samples (see Supporting Information) has shown that no toxic products were formed by the nylon scaffold after a month of exposure to collagenase, the main enzyme of the extracellular matrix (Figure S2), and no ultrastructural changes of the sample. No decrease in cell viability was detected, which means that the resulting materials are unlikely to exert any toxic effects in vivo.

### 3.3. The Morphology of Rat Hippocampal Neurons Grown on Nanostructured Nylon Scaffolds

**Figure 3:**
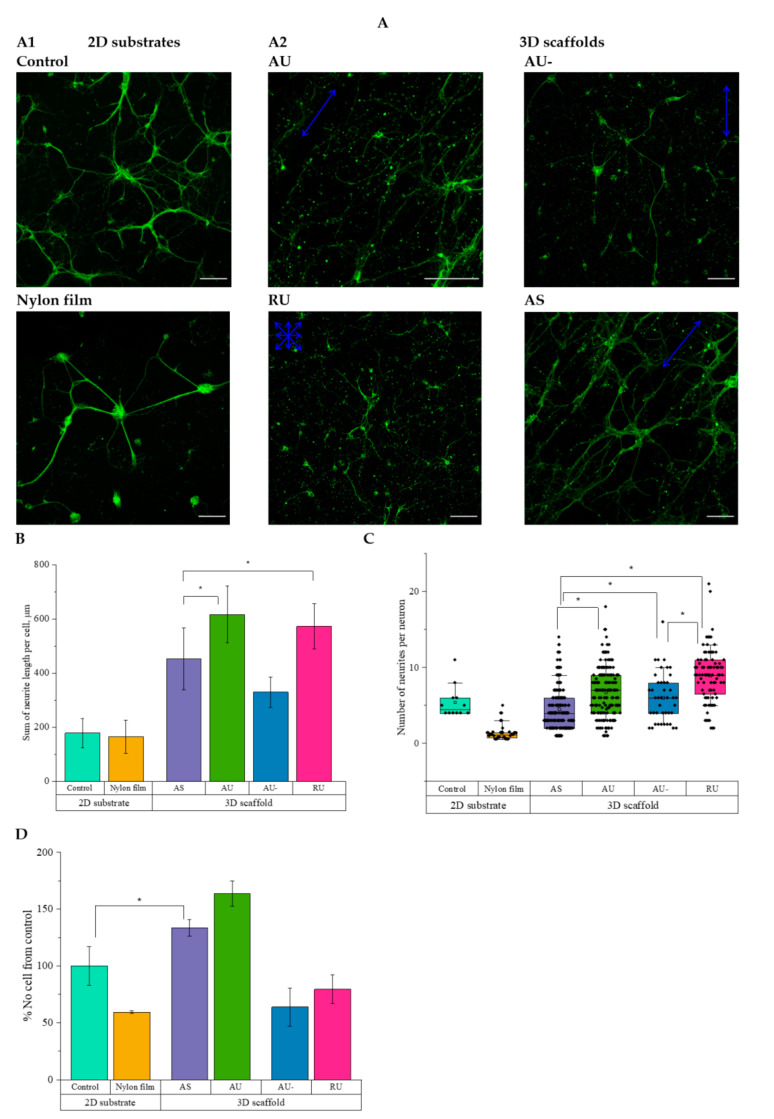
The effect of scaffold surface ultrastructure on the morphology of rat hippocampal neurons. (**A**) Confocal images of neurons growing on different substrates. Control—a poly-D-lysine-coated slide; only AU fibers were not treated with plasma (AU-). Coloring: green—β-tubulin. Blue arrows indicate the direction of fiber packing; intersecting arrows indicate randomly oriented fibers. Scale bar—75 µm. (**B**) Total elongation of neurites per cell during cultivation on 2D and 3D substrates; (**C**) Number of neurites per neuron. The dots correspond to the obtained values; boxes and bars correspond to 25–75% and 10–90% percentiles, consequently; horizontal lines represent median values. (**D**) Number of cells in an area 0.25 mm^2^ as a percentage of control. At least 40 cells were analyzed for each type of substrate. * statistically significant difference, *p* < 0.05.

The bright-field images of neurons growing on the nylon scaffolds are presented in Figure S3, while the confocal imaging data along with their quantitation are shown in Figure 3. As reported earlier, plasma treatment increases the adsorption of proteins on the substrate surface and thereby promotes cell adhesion [32]. Here, we compared the effect of plasma treatment of fibers on the morphology of hippocampal neurons. According to the results of morphometric analysis, the total *elongation* of neurites per cell increased 2-fold, and the number of cells was 2.5-fold higher when neurons were cultured on plasma-treated ultrathin aligned fibers, as compared to the untreated ones (Figure 3B,D). Therefore, in further experiments, only plasma-treated substrates were used.

**Figure 4:**
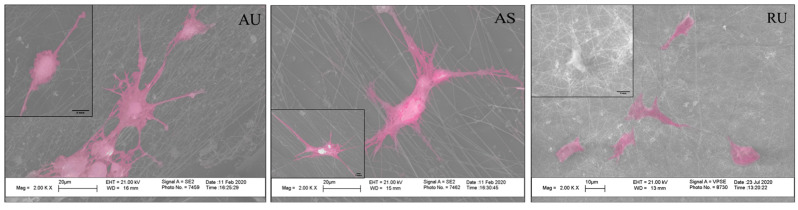
SEM images with color enhancement of rat hippocampal neurons cultured on nanostructured nylon scaffolds for 7 days. Insert—10 µm. The cells are pseudo-colored pink for contrast.

Hippocampal neurons cultured on 2D-substrates showed less total neurite elongation per cell (Figure 3A,B) compared to the 3D-scaffolds. Notably, culturing hippocampal neurons on nylon film consistently led to the formation of neurospheres (Figure 3A1). The greatest total elongation of neurites per cell was observed on ultrathin aligned and random fibers (620 ± 110 and 570 ± 80 μm, respectively; Figure 3B). The details of interaction between neurons and fibers were analyzed using SEM imaging. As seen in Figure 4, the direction of the fibers affected the orientation of the neuronal processes.

**Figure 5:**
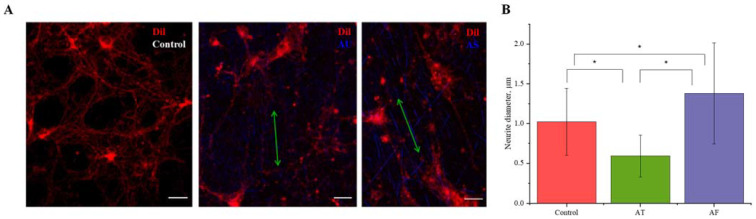
Measurement of neurite diameter using confocal microscopy. (**A**) Confocal image staining of neuronal cell membranes (Dil dye, red). Scale bar—20 µm; (**B**) mean diameter of neurites cultivated on different substrates. * Statistically significant difference, *p* < 0.05.

Cultivating neurons on random ultrathin fibers initiated branching and led to increased numbers of neurites per cell (up to 10) compared to the neurons cultured on aligned fibers: the average numbers of neurites were 4.5 for AS and 6.7 for AU fibers, and 5.6 for control (Figure 3C). To estimate the effect of the fiber diameter on the thickness of the processes of hippocampal neurons, cells were cultured for 7 days on the surface of various substrates, followed by cell membrane staining with the Dil fluorescent probe (Figure 5A and Figure S4). Quantitative analysis showed accelerated growth of neurites with a smaller thickness (0.6 ± 0.3 µm) on ultrathin fibers compared to the control substrate (1.0 ± 0.4 µm), while AS fibers promoted the formation of thicker neurites (1.4 ± 0.6 µm, Figure 5B).

### 3.4. Ultrathin and Submicron Nylon Fibers Facilitate the Nanotopology-Mediated Directional Neurite Growth

**Figure 6:**
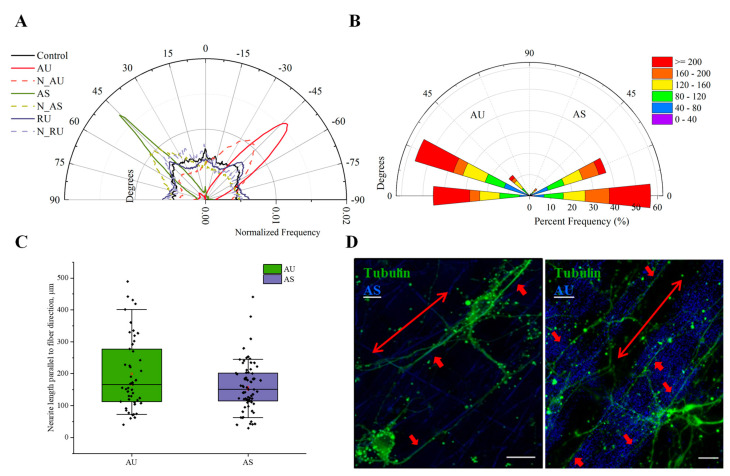
The effect of fiber diameter on nanotopology-mediate guidance of neurites. (**A**) Angular distribution of neurites on control and RU, AU, and AS fibers; (**B**) rose wind plot of the number of neurites of a given length growing at a given angle to the fiber per bin. Each radial distance is indicated by the color and the size of each color represents the percentage of neurite frequency at that distance; (**C**)—average length of neurite parallel to the fiber direction. The dots correspond to the obtained values; boxes and bars correspond to 25–75% and 10–90% percentiles, consequently; horizontal lines represent median values; (**D**) representative confocal images of neurons cultivated on AU and AS fibers (small arrows—neurite parallel to the fiber, large arrows—fiber direction). Scale bar—20 µm.

To characterize the influence of the orientation of nanofibers on the direction of neurite growth, we analyzed angular distribution of neurites in neurons cultivated on nylon scaffolds composed of AS, AU, and RU fibers in vitro (Figure 6). The fibers were visualized in refraction mode at 488 nm (see Materials and Methods). As seen in Figure 6A, the growth of neurites on ultrathin randomly oriented fibers occurred uniformly in all directions, with the range of orientation from −90° to 90°, similar to the control. 

The formation of peaks in the angular distribution of neurites in cells growing on AS and AU fibers, as well as their shift toward the corresponding peaks in the distribution of fibers, suggests that neurites grew in the direction of the fiber orientation (Figure 6A). Comparative analysis of the combined effect of the ultrastructure and substrate anisotropy on the growth of processes showed that a significant part of the neurites (~55%) cultivated on the AS fibers were growing in the direction parallel to them (the angles between the neurites and the fibers did not exceed 10°, Figure 6B–D). At the same time, we observed a large number of neurites with a length of more than 200 μm. Similar results were obtained for ultrathin fibers: about 55% of neurites grew along fibers, and the angles between them and the fibers were in the range of 15–30° (Figure 6B–D). A large proportion of these neurites also had a length exceeding 200 μm. Thus, we have quantitatively shown for the first time that ultrathin fibers effectively guide neurite growth. 

### 3.5. Influence of Matrix Ultrastructure on the Expression of Adhesion Receptors of Hippocampal Neurons

**Figure 7:**
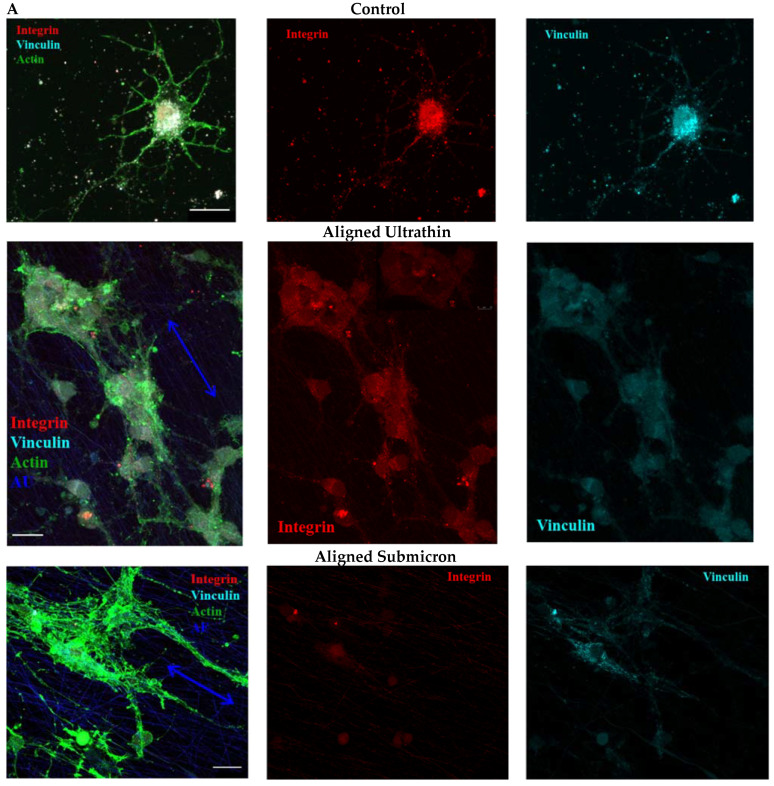

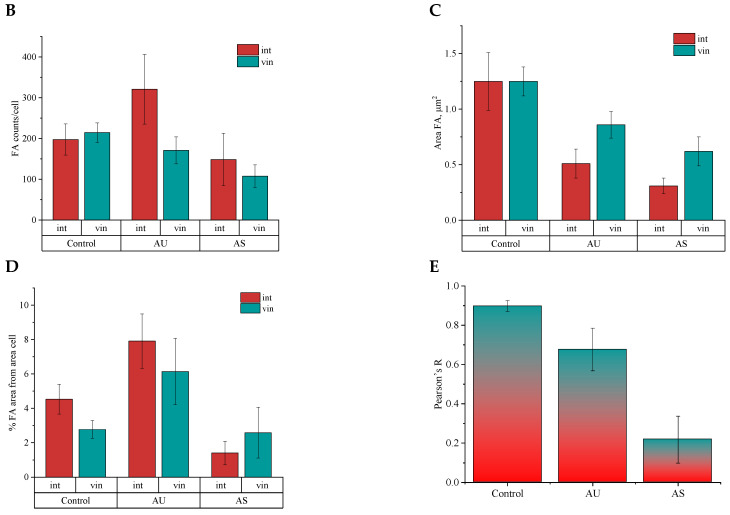
Formation of integrin- and vinculin-containing focal contacts of neurons cultured on glass coated with poly-D-lysine (control) and on ultrathin and submicron nylon fibers. (**A**) Confocal images of cultured neurons stained with antibodies specific for integrin β1 (red) and vinculin (cyan), and with phalloidin for actin network imaging (green). The arrows show the fiber direction; (**B**) number of FAs per cell; (**C**) calculated area of FAs of each kind; (**D**) percentage of FA area relative to the total surface area of the cell; (**E**) colocalization of vinculin- and integrin-positive clusters by Pearson’s R. The number of analyzed cells was at least 50; scale bar—25 μm.

Since the interaction of cells with nylon scaffolds was reported to induce a different type of cell morphology compared to the growth on the glass substrate, it was of interest to evaluate the expression of the focal adhesion (FAs) components, namely, β1-integrin and vinculin, in order to understand the possible mechanisms underlying these changes. To visualize the constituent FAs β1-integrin and vinculin, rat hippocampal neurons were cultured on poly-D-lysine-coated slides and nanofiber scaffolds for 7 days, followed by fixation and staining for immunofluorescence microscopy (Figure 7A). Neurons cultured on glass β1-integrin- and vinculin-positive areas were found to colocalize in small clusters located throughout the surface of the entire cell (Figure 7A,E). This is confirmed not only by comparable numbers of FAs per neuron (Figure 7B), but also by the sizes of the formed clusters (Figure 7C and Figure S5). These data are also consistent with the published data on the interaction of neurons with 2D-substrates [33,34,35].

We found a slight but statistically significant decrease in the number and size of vinculin-containing regions in neuron FAs on AU fibers compared to control (0.9 ± 0.2 μm^2^ and 1.3 ± 0.1, respectively, Figure 7B and Figure S5); however, they occupied a significant part of the cell area (two times more than in the control, Figure 7D). A lower Pearson correlation coefficient (Figure 7E) and, therefore, less pronounced co-localization of the vinculin- and intregrin-stained areas, may be associated with the differences in cluster sizes. The area occupied by vinculin FAs area was twice the size of the area occupied by integrin FAs (Figure 7C). The interaction of neurons with AU fibers led to the formation of a large number of small immature β1-integrin-positive FAs (0.5 ± 0.1 versus 1.3 ± 0.3 μm^2^ in the control; Figure 7B,C and Figure S5), visualized throughout the entire surface of the cells (on the somas and neurites, Figure 7A). The number and size of vinculin-containing clusters of neuronal adhesion on AS fibers were smaller than in the control cells (0.6 ± 0.1 μm^2^, Figure 7B,C and Figure S5), while the area occupied by vinculin FAs was the same (Figure 7D). On AS fibers, vinculin-positive clusters were elongated and oriented along the nanofibers, and they did not colocalize with the integrin-positive regions of FAs (Figure 7E). Integrin staining in neurons growing on AS fibers was significantly altered compared to the control conditions. Staining was diffuse (diffuse puncta in Figure 7 and Figure S3), indicating the formation of nanoclusters, localized predominantly in the cell soma. The number, size, and percentage of the area occupied by β1-integrin-positive clusters decreased compared to the control (Figure 7B,C and Figure S5). In the present study, we did not attempt to perform high-precision measurements of the size of FAs sites; our main goal, similar to the study of Slater et al. [36], was only to detect characteristic changes of these structures when neurons were cultured on different substrates.

### 3.6. Nanofibers Increase the Synapse Number in Neurons

**Figure 8:**
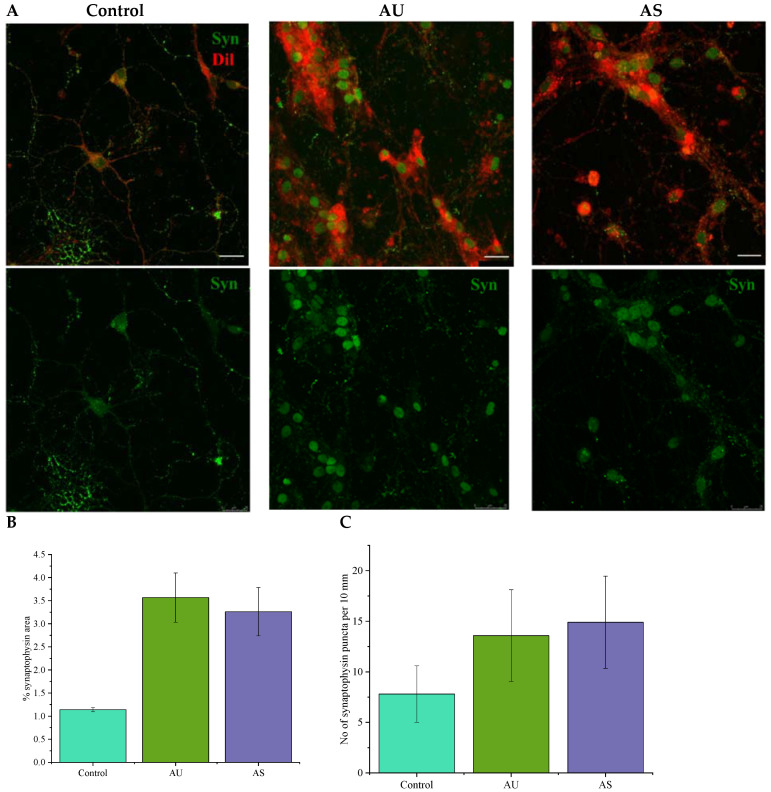
The effect of scaffold structure on the number of synapses formed by neurons. (**A**) Representative images of hippocampal neurons stained for synaptophysin (green) on glass, AU and AS nanofibers (scale bar—25 μm); (**B**) percentage of the area occupied by stained synaptophysin puncta from the area of the cell; (**C**) quantification of synaptophysin puncta on 10 µm neurites.

A question might arise whether cultivation of neurons on nylon ECM-mimetic scaffolds could stimulate the formation of mature synaptic connections and functional neuronal networks. Images of neurons cultured for 7 days on PDL-coated slides or ultrathin and submicron nylon fibers with immunostaining for synaptophysin (syn)-positive puncta are shown in Figure 8. Imaging confirmed the ability of isolated rat hippocampal neurons to form syn-positive contacts on all types of nylon scaffolds. A quantitative comparison in terms of percent-area of synaptophysin staining revealed that experimental scaffolds increased neuronal synaptogenesis compared to the controls (Figure 8B). This is not only due to the increase in mean neurite elongation on 3D-substrates, as shown in Figure 3: culturing neurons on AU and AS fibers increased the density of syn-positive puncta (Figure 8B). Their number on the surface of neurites of contacting neurons doubled when grown on nylon fibers, as seen in Figure 8C.

## 4. Discussion

Various types of nylon have long been used in the industry for fiber fabrication; their physicochemical and mechanical properties are well studied. However, we were the first to use nylon scaffolds with fiber sizes below 100 nm for neural tissue engineering. Production of ultrathin-oriented nylon scaffolds has made it possible to study guided neurite growth on fibers with characteristic scales mimicking the architecture of ECL. It should be noted that somewhat more prominent alignment of thicker fibers has been reported earlier [8,13,17,20]. This can be explained by the relatively small diameter of the fibers we studied; according to the published data [8], the thinner the fibers, the less the degree of their alignment.

The most important characteristic of any implant is its biocompatibility. Studying the viability of neuron-like IMR-32 human neuroblastoma cells on nylon fibers revealed no toxic effects (Figure 2A). All types of nylon substrates showed significant increase in cell counts over time compared to controls (Figure 2B). This means that, first, the fibers were not toxic to cells, and second, the high surface area-to-volume ratio and the porous structure of nanofibers might increase the contact area between cells and fibers [37]. It is important to note that the rates of cell proliferation observed on nylon scaffolds did not depend on the degree of fiber orientation. It was previously proposed that the effect of fiber orientation on cell proliferation was cell-specific. For example, PC12 cells proliferated more intensively on oriented nanofibers as compared to randomly oriented ones [10]. Similar results were obtained for Schwann cells when cultured on aligned and randomly oriented PLGA nanofibers [38]. However, no statistical differences in viability were found between mouse neural stem cells cultured on aligned and random polyphenylene sulfone fibers with a diameter of 735 nm [39]. Similarly, the number of neonatal mouse cerebellar C17.2 stem cells grown on PLLA fibers was shown to be independent of whether aligned or randomly oriented fibers were used; however, cell proliferation was significantly reduced on the fibers smaller (350 nm) or larger (1150 nm) than 500 nm [40]. In the present work, a decrease in the fiber diameter to 60 nm did not suppress cell growth. 

It is known that to stimulate cell adhesion in the absence of ECM proteins or other contact control guidance cues, the surface of synthetic nanofiber matrices is best coated with adhesion promoting agents such as poly-D-lysine, polyethyleneimine, collagen, or laminin (Table 1) [41,42]. Since interactions and contact formation of neurons with the substrate are determined by both biochemical and mechanical stimuli, we investigated the influence of the chemical properties and topographic features of the substrate surface on the morphology of neurons. We showed that in the first 24 h the cells adhered and spread on all types of nylon fibers (Figure S3). When cultured on glass, neurites grew in different directions, while neurons cultured on AU and AS scaffolds had long neurites directed along the fibers. The highest total elongation of neurites per cell was observed on ultrathin aligned and random fibers (Figure 3B). Thus, the influence of fiber topography on the morphology (length and direction of neurite growth) and differentiation of nerve cells is evident. According to the published data (Table 1), the maximum growth of neurites was previously observed on oriented fibers 500–1000 nm in diameter. Structural components with the thickness of 500 nm turned out to be the most optimal, as assessed by the neurite growth rate and directionality [43]. At the same time, there’s hardly any published reports about the growth of neurons on scaffolds with ECM-like architecture and fiber diameter of ~10–300 nm. We have managed to fabricate 3D nylon scaffolds with a unique nanotopology, and this is the first demonstration that ultrathin fibers promote the elongation of the neurites of hippocampal neurons even more efficiently than those with a diameter of 300 nm.

We show that AU fibers initiated an accelerated growth of neurites with a smaller thickness compared to the AS and the control substrate (Figure 5E). Similar results were previously obtained by Daud et al. [15] for the neurite growth on PCL fibers with 1–8 μm diameter. Tomba et al. [44] found a relationship between neurite thickness, actin wave production rate, and the rate of neurite elongation. They concluded that thin neurites elongated faster, which is consistent with our data on the elongation rate during neuronal growth on AU fibers. However, the thickness of neurites does not affect the innervation process and can be regulated by the formation of neuromuscular contacts [45].

It should be noted once again that scaffolds for neurosurgery with fiber diameters less than 200 nm have not been previously studied. We can only point to the work of Ghasemi-Mobarakeh et al. [8], who showed the oriented growth of neural stem cells on aligned nanofibers PCL/gelatin with a diameter of 190 nm; however, there was no quantitative analysis of morphological parameters (Table 1). In other studies using submicron fibers with a diameter of <500 nm (see Table 1), directional growth of neurites upon interaction with thin fibers was not recorded.

To address the legitimate question of whether scaffolds made of oriented ultrathin nylon fibers have any advantages in practical use, we tried to compare the morphometric parameters of neurons registered in our study with the previously published data (Table 1). Unfortunately, the use of different cell models and cultivation conditions in the published research did not allow for an adequate comparative analysis. Nevertheless, judging by comparison of the neurite elongation values, it can be concluded that ultrathin nylon fibers are not inferior to, and in many cases are superior to electrospun fibers obtained from FDA-approved materials such as PLLA, PCL, and PLGA (Table 1). In addition, given the biocompatibility, controlled biodegradation and low cost of nylon-4,6, as well as the absence of the need to treat these substrates with adhesive agents, nylon scaffolds made of ultrathin and submicron fibers can be considered highly applicable for neural tissue engineering.

Cell-substrate interactions are regulated by complex mechanisms and are strongly influenced by nanoscale structures. Morphological changes in neurons (microscale process) can be governed by topographic signals that are an order of magnitude smaller than the cell size, up to 10 nm [46,47]. The sensitivity of neurons to topographic signals up to 500 nm was shown to be associated with the FAs size, which ranges from 5 to 200 nm and up to 10 μm for mature forms [48,49,50]. Changede et al. [50] found single matrix fibers ≤30 nm to be unable to support FA formation and downstream signaling. Stable nanoclusters could be assembled when ligands were 40–60 nm apart [33,51]. In contrast to large FAs formed by cells on glass, cell adhesion to the matrix in vivo can form pointwise, possibly due to the presence of ECM fibers 5–20 nm in diameter. It should be emphasized that, in general, the formation of cell-matrix adhesions on fibrous substrates and the impact of nanoscale geometric cues on adhesion size and cell morphology are poorly understood [34,51]. Here, we tried to expand our understanding of the interaction of neurons with an ECM-like matrix composed of thin fibers and to evaluate the expression of FAs components, namely, β1-integrin and vinculin.

The role of vinculin in the cell sensing of nanoscale topography and geometry has been studied in [52,53]. Vinculin was shown to be involved in the formation of large FAs (>1 μm^2^) as well as emerging focal contacts; however, in nanoscale models, vinculin recruitment is reduced because of a decrease in FA size, which in turn suppresses the assembly of stable actin cytoskeleton. It was concluded that nanotopography sensing by cells did not mediate the recruitment of vinculin, but it was mediated through the micro-scale organization of the actin network. Our results revealed a decrease in the number and size of vinculin-containing regions of neuronal FAs on AU fibers, which occupy a significant part of the cell area as compared to the control (Figure 7B and Figure S5). β1-Integrin-positive FAs were visualized throughout the entire surface of the neuron (Figure 7A) cultured on ultrathin fibers. This indicates that a large number of immature FAs arising on AU nanofibers, despite their small size, promoted recruitment of vinculin and assembly of the actin cytoskeleton. As seen in Figure S5, actin fibers were sparser and radially directed toward the edges of the cell, in contrast to the control cells, which showed large adhesions and actin stress fibers at the cell periphery.

The interaction of neurons with AS fibers led to the formation of elongated vinculin-containing clusters oriented along the nanofibers; however, their number and size were smaller compared to the control cells. In this case, area occupied by vinculin FAs was not different from control cells values; thus, there was no significant suppression of vinculin recruitment. Moreover, multiple actin stress fibers were found to form in the cells (Figure 7 and Figure S5). Integrin staining was diffuse, indicating the formation of nanoclusters, localized predominantly in the soma. The number, size and percentage of the area occupied by β1-integrin-positive clusters decreased compared to the control (Figure 7B,C and Figure S5). Our data showing a decrease in the FAs size during the interaction of cells with nanoscale substrates are consistent with the results of Slater et al. [54]. They showed that limiting adhesion site growth to small, immature adhesions using sub-100 nm patterns induced cells to form a significantly higher number of smaller, more densely packed adhesions that displayed few interactions with actin stress fibers. Endothelial cells exhibiting these traits were characterized by highly dynamic fluctuations in spreading and active migration. Increase in the size of the patterns correlated with increase in the size, while their number decreased and the ability of cells to migrate was reduced. A similar decrease in the size of cell FAs and increased cell migration upon interaction with a nanoscale substrate had been shown in other studies [36,55]. Above, we showed the formation of neurospheres on ultrathin fibers, which may be an indication of enhanced migration of hippocampal neurons along them (Figure S6). Enhanced neuritogenesis (Figure 3) by neurons growing on AU fibers may also be associated with the formation of immature FAs. The same assumption was made by Schulte et al. [56]. However, the architecture (non-fibrillar structures) and the size of nanopatterns on the substrates used in the aforementioned studies differs significantly from those of ECM.

A noticeable decrease in β1-integrin subunits on the surface of the cells cultured on AS fibers as compared to AU fibers (Figure 7) may be due to the participation of alternative integrin subunits in FAs assembly (e.g., β3 subunits). As shown by Schaufler et al. [57], activation of αvβ3 integrin by ligand binding is dispensable for initial adhesion and spreading, but is necessary for the formation of stable AFs. In addition, αvβ3 integrin binding enables structural reinforcement of the integrin-actin linkage through the recruitment of vinculin; and enhanced expression of β3 integrin can compensate for the loss of β1 integrin [58,59,60]. At the same time, β1-expressing cells are less sensitive to the dimensions of nanofibers compared to β3-expressing cells [61]. It was suggested that β1- and β3-integrin expressing cells could regulate the assembly of their actin cytoskeleton through different pathways, despite the fact that the heterodimers they formed recruited similar adapter proteins [53]. Thus, the formation of FAs with the participation of β3-integrin does not contradict the published data and is consistent with the observed intense formation of actin bundles in neurons cultured on AS fibers. However, the assumption that both the chemical nature of the material and the substrate nanotopology can affect the FAs assembly through certain integrin subunits, requires additional verification.

Culturing neurons on aligned ultrathin and submicron fibers increased the density of synaptophysin-positive puncta on the surface of neurites of contacting neurons (Figure 8). Thus, both types of aligned nylon fibers, with the average diameter of 60 nm and 300 nm, not only stimulate the directed growth of neurites, but also promote synaptogenesis and formation of neuronal connections, which is critical for the regeneration of functional nerve tissue.

It could be assumed that the significant elongation of neurites observed during cultivation of neurons on AU fibers is associated with the formation of a high number of small “immature” dynamic FAs which contribute to the rapid rearrangement of actin structures and, ultimately, the growth of neurites. It is very likely that in vivo the role of collagen fibers < 100 nm in diameter is to initiate migration and accelerated cell growth, while larger fibers provide directional cues. These phenomena can be used to create ECM-mimetic scaffolds with structural unit sizes < 100 nm; these scaffolds could serve as novel materials with superior biological properties for regenerative medicine applications.

## 5. Conclusions

We present for the first time nanoscaffolds composed of polymeric fibers with diameters less than 100 nm, which can efficiently control neurite growth. Scaffolds formed by 60 nm diameter ultrathin nylon nanofibers with both random and aligned fiber orientation were fabricated by electrospinning. In order to reveal the effects of the nanoscale structure, we used scaffolds consisting of thicker nylon nanofibers 300 nm for comparison. Analysis of the growth of rat hippocampal neurons on novel scaffolds revealed significant advantages of nanoscale nylon scaffolds. They appeared to be more favorable for cell proliferation, increased elongation of neurites, and provided guidance for neurite outgrowth, which is the first time such an effect has been reported. Distinct mechanisms of neuronal adhesion to ultrathin and submicron nylon fibers have been discovered. Our observation that neurons form small immature β1-integrin-positive FAs on ultrathin fibers, in contrast to submicron scaffolds, suggests that scaffold nanotopology may affect the assembly of FA by involving various integrin subunits. Other experiments demonstrated comparably good performance of both ultrathin and submicron scaffolds in terms of biocompatibility, axon alignment, and synaptogenesis. In conclusion, our results suggest that nanostructures with a characteristic scale below 100 nm, similar to the size of ECM structures, are able to serve as efficient scaffolds with superior properties for nerve tissue regeneration.

## Figures and Tables

**Table 1:** Summary of the nanofiber size effects on neuron morphology.

Scaffold Material/Cell Model	Diameter Fiber, nm	Cell Response/Behavior	Reference
**Fiber diameter < 500 nm**			
PCL, PCL/gelatin + serum protein // C17.2	113 ± 33, 189 ± 56, 431 ± 118	Oriented growth of neurites on 189 ± 56 nm fibers	Ghasemi-Mobarakeh et al., 2008 [8]
PCL + PLO and laminin // ANSCs	260, 480 and 930	Cell elongation and enhanced differentiation on 480 nm aligned fibers	Lim et al., 2010 [9]
PLGA without treatment / PC12	400–500	ANL - 160 μm in 5 days	Kim et al., 2016 [10].
PLLA/PC12	269.30 ± 4.27	ANL - 21.29 μm in 24 h	Lü et al., 2017 [11]
PLLA + fibronectin // Astrocytes and neurons from DRG	386/808	No directional alignment along 386 nm fibers. ANL parallel to 386 nm fibers - 220 µm, 808 nm - 320 µm in 4 days	Johnson et al., 2019 [12]
**Fiber diameter > 500 nm**			
	Diameter fiber, μm		
PLLA + laminin // neurons from DRG	2.19 ± 0.19 2.31 ± 0.06 2.44 ± 0.07	MNL - 476.3 - 501.7 μm in 4 days	D’Amato et al., 2019 [13]
PLLA + DETA, AEE, GRGDS and treatment O_2_ plasma // chicken DRG	0.9–1.3	MNL increases from 844 to 1651 μm in 5 days, depending on the treatment of fibers	Schaub et al., 2015 [14]
PCL // NG108-15	1.02 ± 0.05 5.08 ± 0.130 8.07 ± 0.07	MNL decreases from 142.36 to 61.83 μm in 4 days with decreasing fiber diameter. Oriented growth of cells	Daud et al., 2012 [15]
PLLA + serum protein // DRG	1.325 ± 0.383 0.759 ± 0.179 0.293 ± 0.65	MNL decreases from 1400 to 1000 μm in 5 days with decreasing fiber diameter. No oriented growth of cells on 293 nm fibers	Wang et al., 2010 [16]
Gelatin/PCL (70:30) // NG108-15	0.63 ± 0.13	ANL ~ 120 µm in 5 days. Oriented growth of cells	Soliman et al., 2018 [17]
PLGA and PLGA-MWCNTs-COOH + plasma treatment // DRG	0.913 ± 0.185 0.768 ± 0.127 0.708 ± 0.142 0.631 ± 0.94	ANL increases from 19.23 to 78.27 μm in 3 days with increasing CNTs content	Wang et al., 2017 [18]
PLGA + laminin // ND7/23	5.73 ± 0.57 0.74 ± 0.18	ANL on 5 μm fibers ~ 175 μm (160% of control), on 0.74 μm fibers ~ 110 μm (105%). Oriented growth of cells	Binder et al., 2013 [19]
PPy-PLLA + laminin and collagen // PC12	~0.8	ANL on random and aligned fibers - 65.44 and 114.73 μm in 3 days. Oriented growth of cells	Zou et al., 2016 [20]
Silk + reduced graphene paper // SH-SY5Y	0.5–0.55	ANL on random and aligned fibers - ~350 and 1200 μm in 10 days. Oriented growth of cells	Qing et al., 2018 [21]

Abbreviations: ANL—average neurite length; AEE—2-(2-aminoethoxy)ethanol; ANSCs—Multipotent adult neural stem cells; DRG—dorsal root ganglion; DETA—diethylenetriamine; GRGDS—cell adhesion peptide); MNL—maximum neurite length; MWCNTs-COOH—walled carbon nanotubes–COOH; PCL—polycaprolactone; PLGA—poly(lactic-co-glycolic acid); PLLA—poly(L-lactic acid); PLO—poly(L-ornithine).

## Data Availability

The data presented in this study are available in Supplementary Information.

## Supplementary Materials

The following are available online at https://www.mdpi.com/2079-4991/11/2/516/s1. Figure S1. In vitro study of biocompatibility of the experimental scaffolds. Representative confocal images of IMR-32 cell viability when cultured on AU and AS fibers for 3 days. Coloring: green—calcein-AM, red—PI. Scale bar—100 µm. Figure S2: In vitro degradation of nylon fiber nanomats. Figure S3: Phase contrast microscopic images of rat hippocampal neurons showing the time course of their growth on nylon scaffolds. Figure S4. Measurement of neurite diameter using confocal microscopy. Confocal image staining of neuronal cell membranes (Dil dye, red). Scale bar—20 µm. Figure S5: Representative photomicrographs demonstrating the differences in the size of FAs formed by neurons when cultured on different types of substrates. Figure S6: Formation of neurospheres on oriented ultrathin fibers.
