# Supplementary material for: ECM-Mimetic Nylon Nanofiber Scaffolds for Neurite Growth Guidance
Source: Nanomaterials (Basel). 2021 Feb 18;11(2):516. doi: 10.3390/nano11020516 (PMC7922859; doi:10.3390/nano11020516)
Supplement: Supplementary file 1 [file nanomaterials-11-00516-s001.pdf]

Supporting Information

<table>
<tr><td>Control
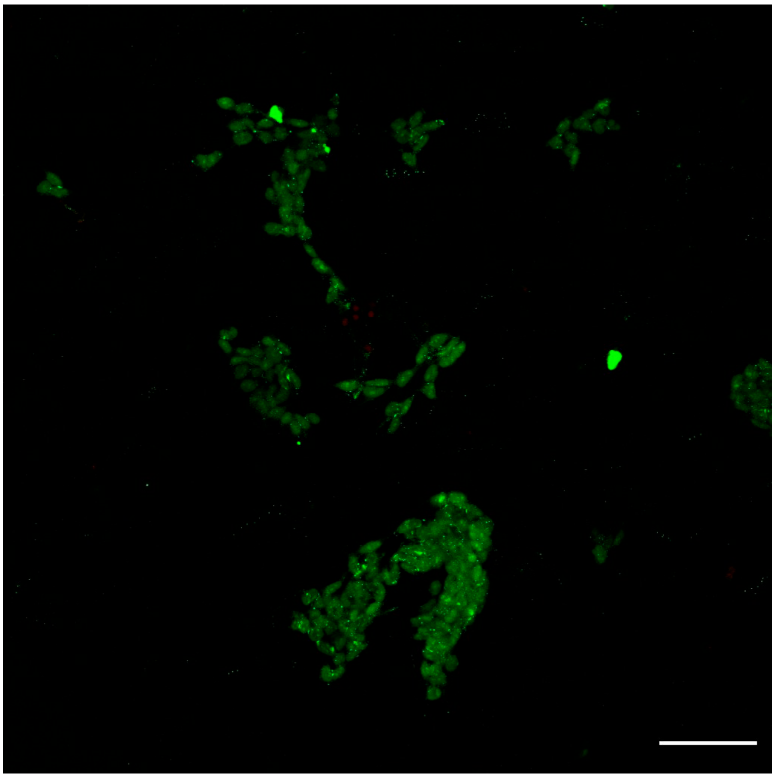</td></tr>
<tr><td>AU
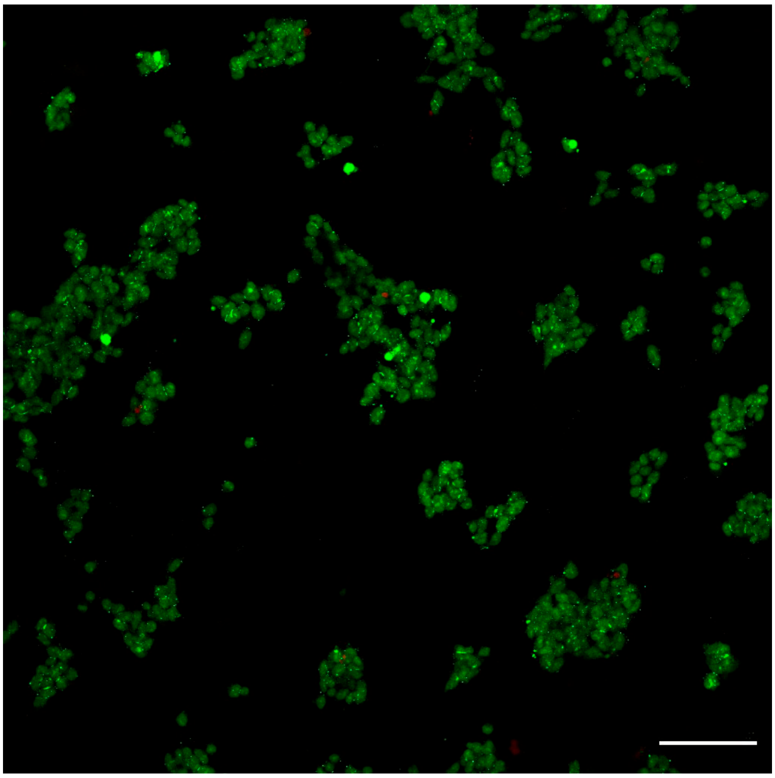</td></tr>
<tr><td>AS</td></tr>
</table>

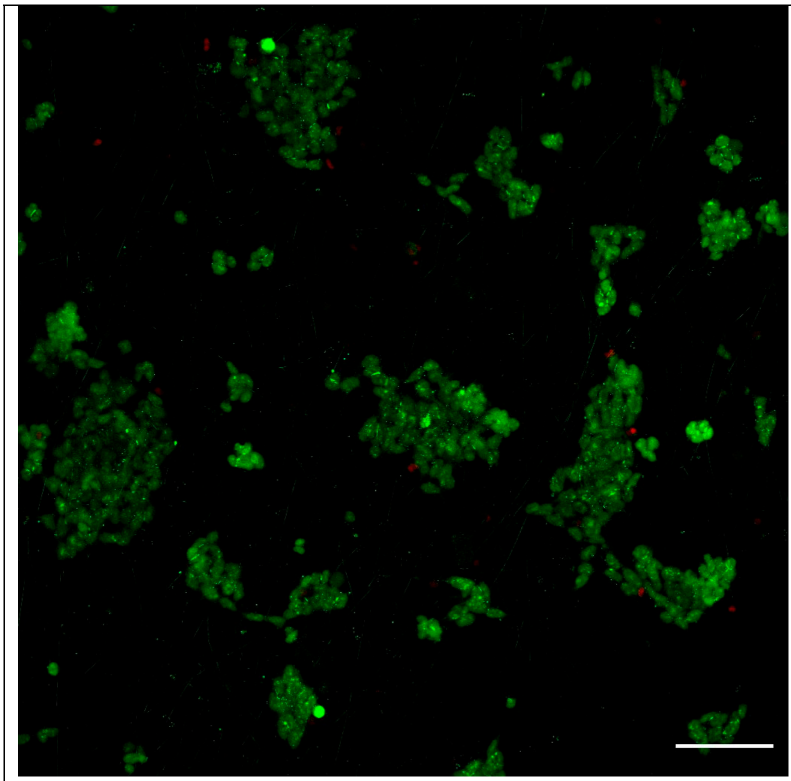

Figure S1. In vitro study of biocompatibility of the experimental scaffolds. Representative confocal images of IMR-32 cell viability when cultured on AU and AS fibers for 3 days. Coloring: green – calcein-AM, red – PI. Scale bar – 100 µm.

Analysis of enzymatic degradation of nylon nanofibers and cytotoxicity of degradation products.

As an additional criterion for the biocompatibility of scaffolds made of nylon-4,6 nanofibers, we studied degradation of nanofibers by collagenase, the main enzyme of the extracellular matrix (**Figure S1**). The degree of toxicity of the products of enzymatic degradation of nylon fiber scaffolds was also evaluated.

A

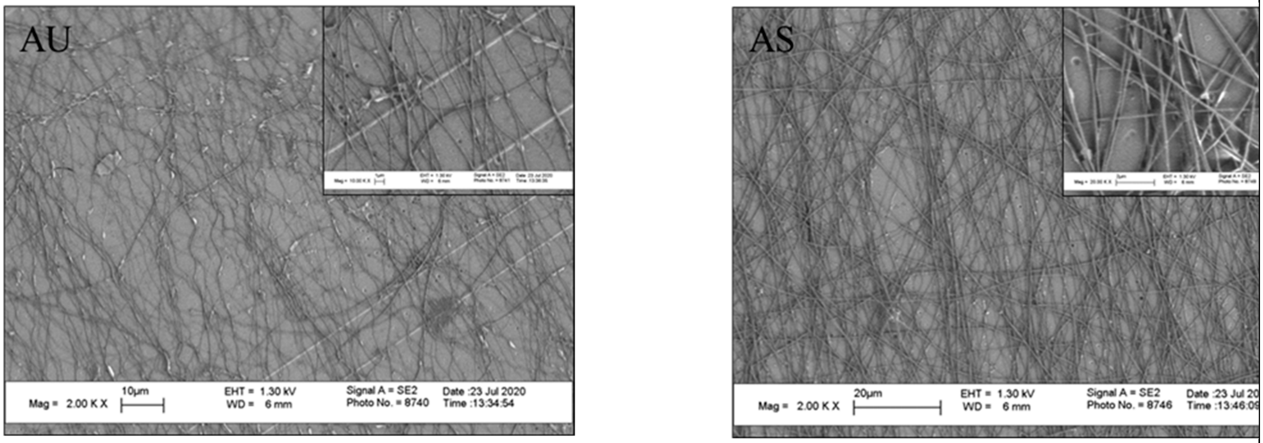

B

Figure S2. In vitro degradation of nylon

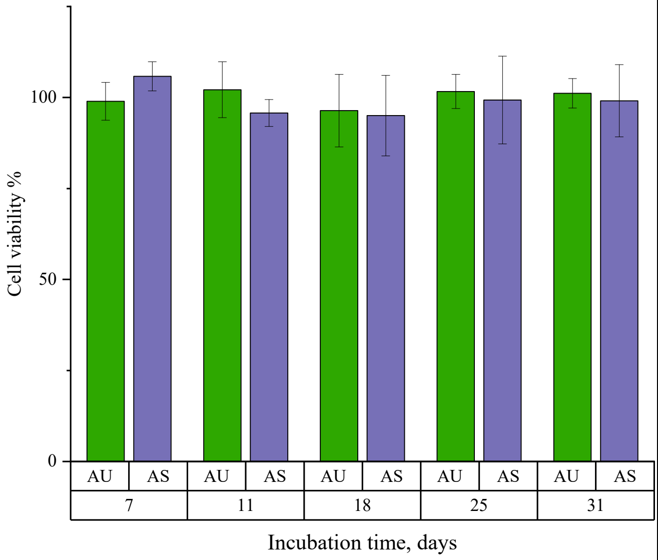

fiber nanomats. A – Electron microscopic studies of the degradation of nanostructured scaffolds under the action of collagenase for 1 month. B - Influence of the products of nanofiber degradation by collagenase on the viability of IMR-32 cells. Cell viability was calculated as percentage of viable cells exposed to the collagenase solution in which the scaffolds had been incubated.

The SEM images showed that after incubation of the scaffolds in collagenase solution for a month, no significant destruction of nylon nanofibers occurred; at this point in time, no swelling of the polymer fibers has yet been observed. This means that these scaffolds are suitable for long-term operation. The results of the viability tests of human neuroblastoma IMR-32 cells incubated with collagenase solutions after incubation with nylon fibers are shown in **Figure S1B**.

No toxic effects or decrease in cell viability were observed when cells were subjected to supernatants containing potential products of scaffold degradation by the main ECM enzyme collagenase, which was inactivated prior to the essay (as described in the Methods section 2.9)

Control	AU	AS	RU
**1st day**			
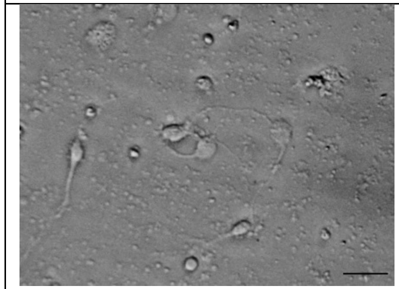	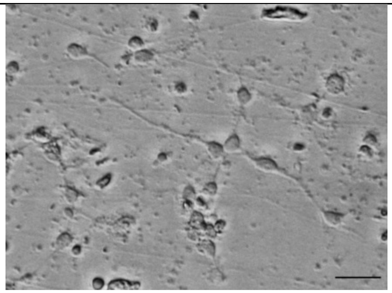	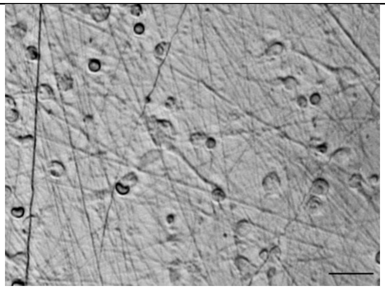	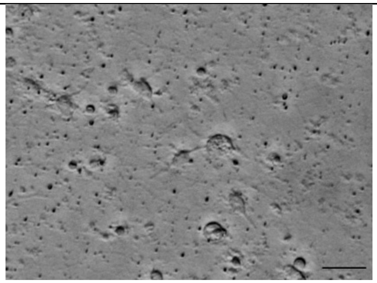
**2nd day**			
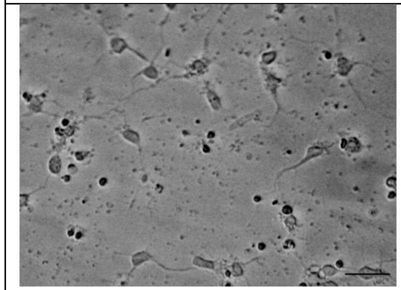	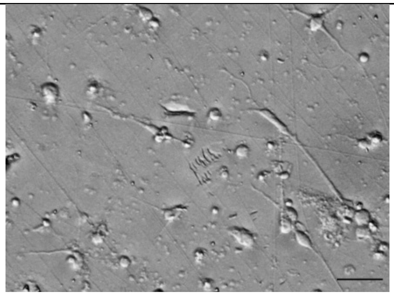	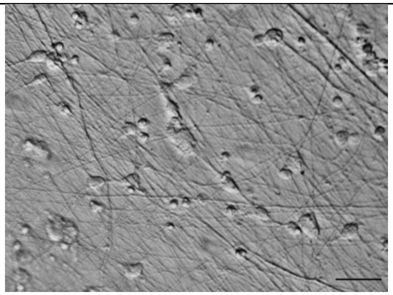	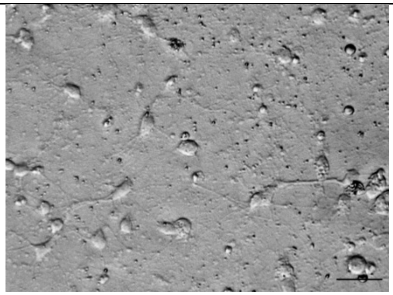
**5th day**			
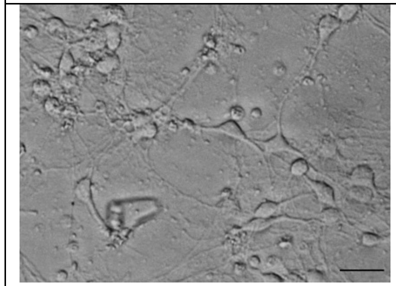	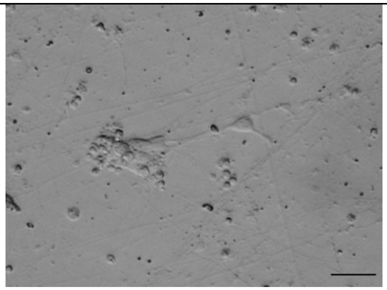	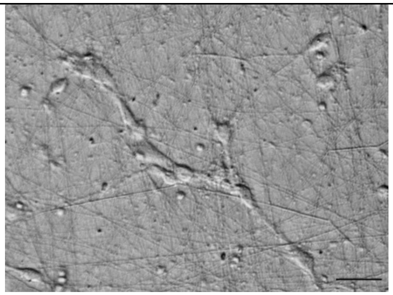	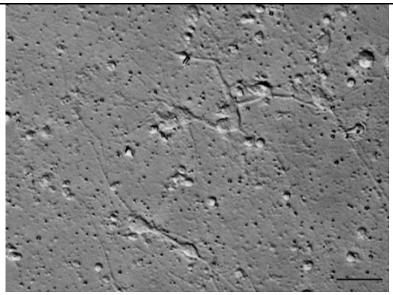

7th day

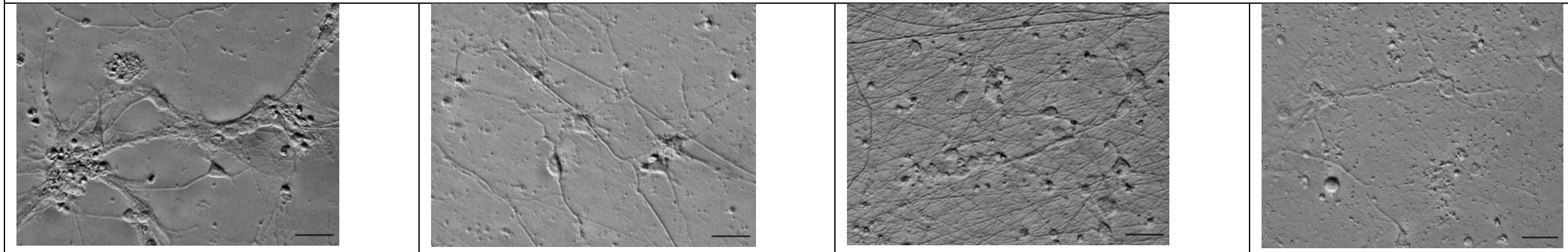

Figure S3. Phase contrast microscopic images of rat hippocampal neurons showing the time course of their growth on nylon scaffolds. Control (PDL) - glass coated with poly-D-lysine; AU – alignated ultrathin fibers, AS - alignated submicron fibers, RU - randomly oriented ultrathin fibers; scale bar – 50 μm.

Control<br>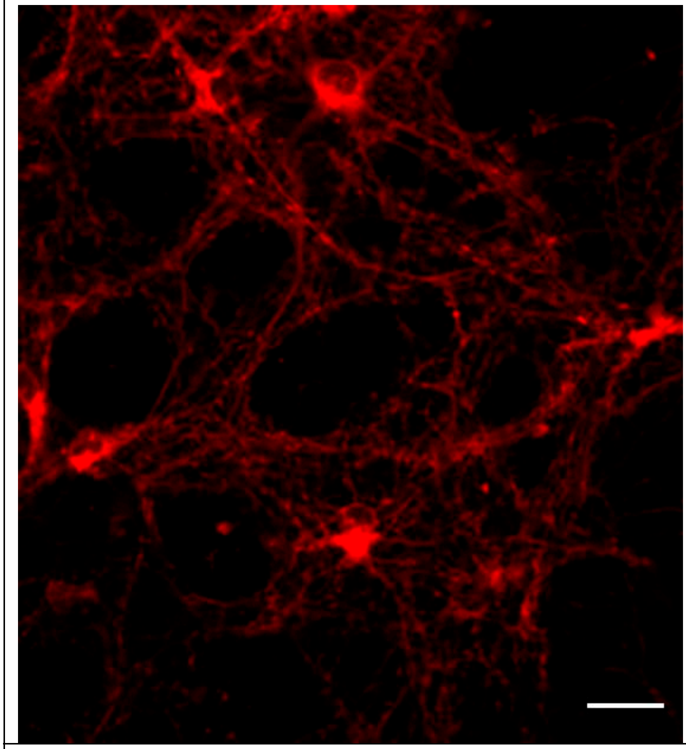	
AU<br>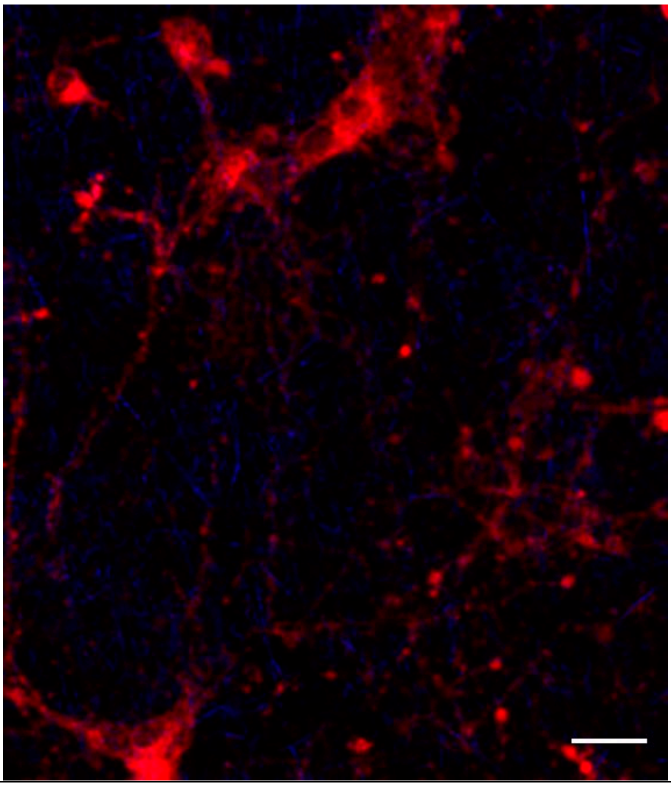	
AS	

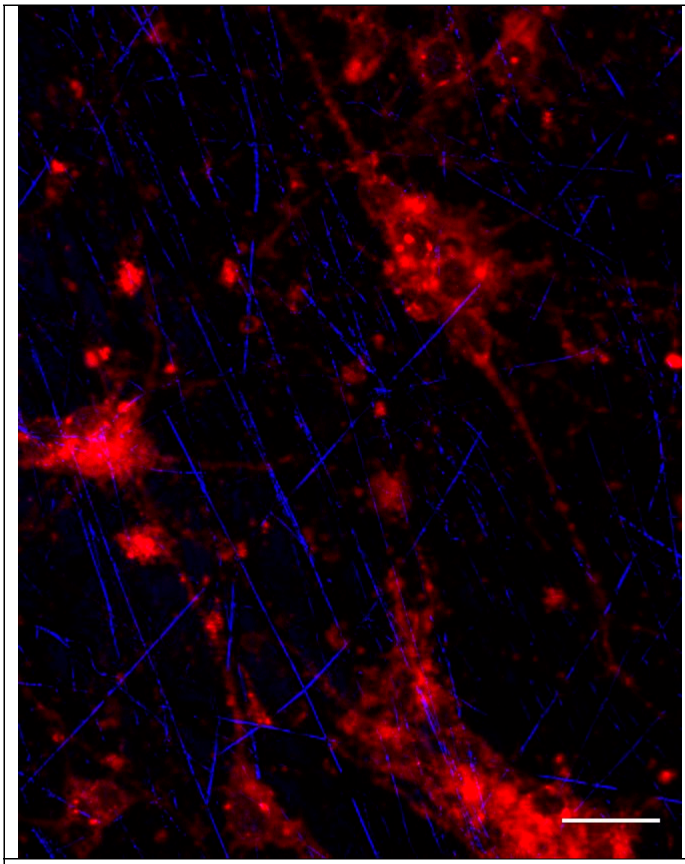

Figure S4. Measurement of neurite diameter using confocal microscopy. Confocal image staining of neuronal cell membranes (Dil dye, red). Scale bar – 20 μm.

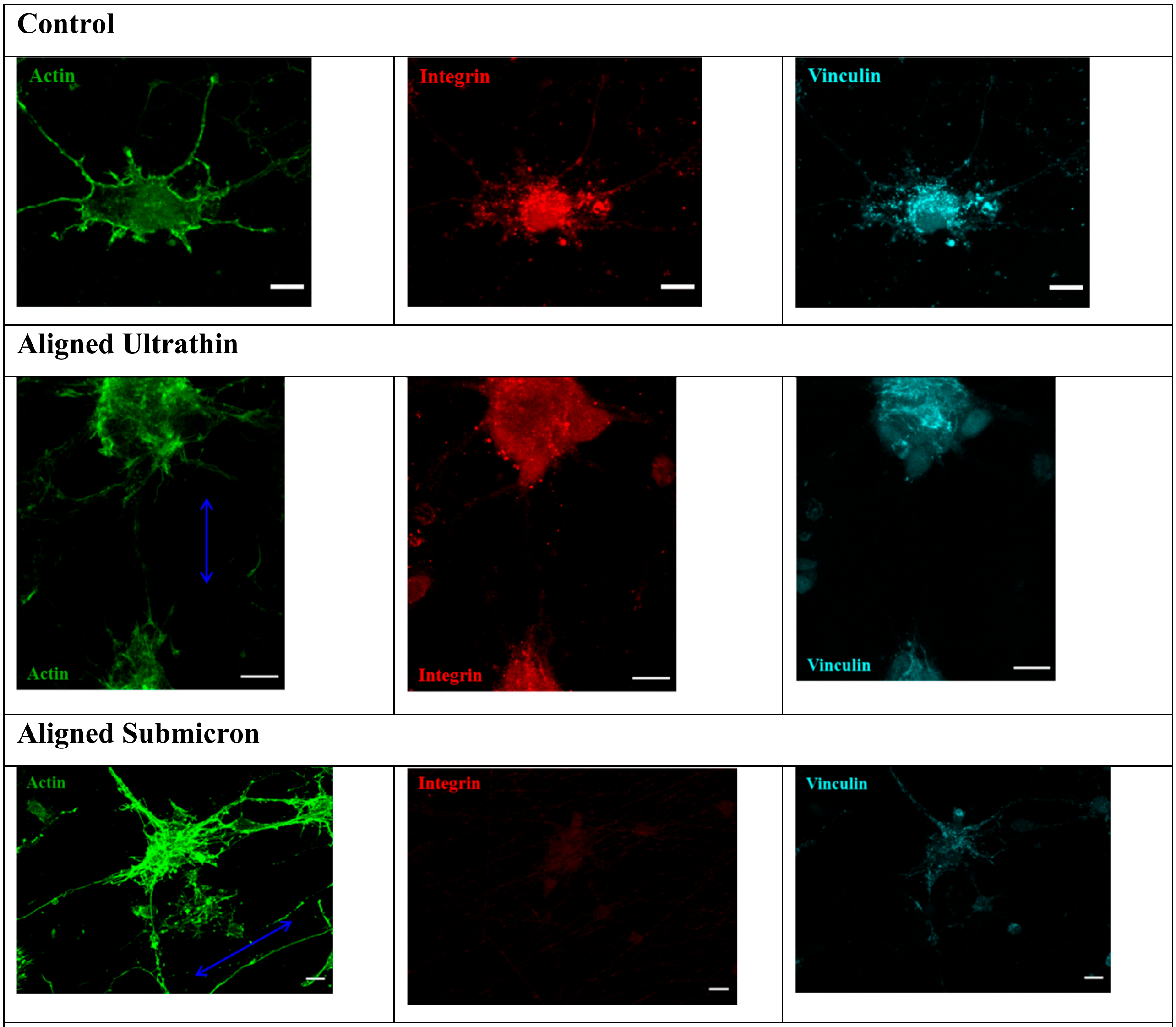

Figure S5. Representative photomicrographs demonstrating the differences in the size of FAs formed by neurons when cultured on different types of substrates. The arrows show the fiber packing direction; scale bar – 15 μm.

A B

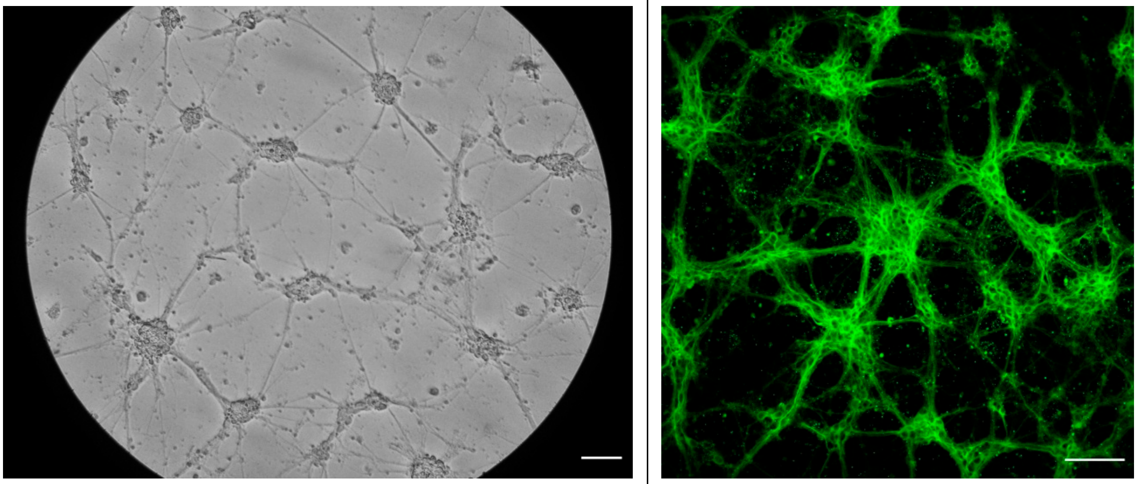

Figure S6. Formation of neurospheres on oriented ultrathin fibers. A – Transmission light image of neurospheres of rat hippocampal neuron cultured on ultrathin fibers; scale bar – 100 μm. B - Confocal images of neurospheres stained β3-tubulin (green); scale bar – 75 μm.
